# Measuring the Quality of Clinical Skills Mobile Apps for Student Learning: Systematic Search, Analysis, and Comparison of Two Measurement Scales

**DOI:** 10.2196/25377

**Published:** 2021-04-23

**Authors:** Tehmina Gladman, Grace Tylee, Steve Gallagher, Jonathan Mair, Rebecca Grainger

**Affiliations:** 1 Education Unit University of Otago Wellington Wellington New Zealand; 2 Hutt Valley District Health Board Lower Hutt New Zealand; 3 Education Unit Dunedin School of Medicine University of Otago Dunedin New Zealand

**Keywords:** mobile apps, MARS, MARuL, medical education, app review, mobile phone

## Abstract

**Background:**

Mobile apps are widely used in health professions, which increases the need for simple methods to determine the quality of apps. In particular, teachers need the ability to curate high-quality mobile apps for student learning.

**Objective:**

This study aims to systematically search for and evaluate the quality of clinical skills mobile apps as learning tools. The quality of apps meeting the specified criteria was evaluated using two measures—the widely used Mobile App Rating Scale (MARS), which measures general app quality, and the Mobile App Rubric for Learning (MARuL), a recently developed instrument that measures the value of apps for student learning—to assess whether MARuL is more effective than MARS in identifying high-quality apps for learning.

**Methods:**

Two mobile app stores were systematically searched using clinical skills terms commonly found in medical education and apps meeting the criteria identified using an approach based on PRISMA (Preferred Reporting Items for Systematic Reviews and Meta-Analyses) guidelines. A total of 9 apps were identified during the screening process. The apps were rated independently by 2 reviewers using MARS and MARuL.

**Results:**

The intraclass correlation coefficients (ICCs) for the 2 raters using MARS and MARuL were the same (MARS ICC [two-way]=0.68; *P*<.001 and MARuL ICC [two-way]=0.68; *P*<.001). Of the 9 apps, Geeky Medics-OSCE revision (MARS Android=3.74; MARS iOS=3.68; MARuL Android=75; and MARuL iOS=73) and OSCE PASS: Medical Revision (MARS Android=3.79; MARS iOS=3.71; MARuL Android=69; and MARuL iOS=73) scored highly on both measures of app quality and for both Android and iOS. Both measures also showed agreement for the lowest rated app, Patient Education Institute (MARS Android=2.21; MARS iOS=2.11; MARuL Android=18; and MARuL iOS=21.5), which had the lowest scores in all categories except information (MARS) and professional (MARuL) in both operating systems. MARS and MARuL were both able to differentiate between the highest and lowest quality apps; however, MARuL was better able to differentiate apps based on teaching and learning quality.

**Conclusions:**

This systematic search and rating of clinical skills apps for learning found that the quality of apps was highly variable. However, 2 apps—Geeky Medics-OSCE revision and OSCE PASS: Medical Revision—rated highly for both versions and with both quality measures. MARS and MARuL showed similar abilities to differentiate the quality of the 9 apps. However, MARuL’s incorporation of teaching and learning elements as part of a multidimensional measure of quality may make it more appropriate for use with apps focused on teaching and learning, whereas MARS’s more general rating of quality may be more appropriate for health apps targeting a general health audience. Ratings of the 9 apps by both measures also highlighted the variable quality of clinical skills mobile apps for learning.

## Introduction

### Background

Mobile apps are widely used by health care professionals and have been shown to improve documentation, workflows, access to information, and clinical decision support [1]. Apps can be found from web-based vendors (app stores), web-based repositories (app repositories, online communities, and news stories), and peer-reviewed literature [2]. A recently published framework for finding apps [3] recommends peer-reviewed literature as the first source of information on quality apps. This nascent body of literature includes high-quality evaluations of single apps and systematic searches of app stores for apps, often including the appraisal of app quality. There is an emerging literature on systematic app store searches for apps to support clinical care [4-10] and the development of instruments for assessing app quality, such as the Mobile App Rating Scale (MARS) [11]. Although mobile apps are now widely accessible and being implemented in clinical care, the role of mobile apps in medical education is less well evaluated.

Mobile app use for teaching and learning has been an area of exploration since the first smartphones became available, and there has been growing use since then [12]. With this increased use, some studies have aimed to determine the characteristics of mobile apps that best contribute to student learning [12,13]. The app characteristics that users identify as best-promoting self-regulated and deep learning include perceived usefulness, perceived satisfaction, and interactivity [13,14]. Although frameworks for implementing mobile technology in medical education have been proposed [15], the evaluation of mobile technology use among medical students is largely limited to surveys evaluating types of apps used and extent of use [16-19] and barriers and facilitators to the use of mobile devices [20,21]. To date, there have not been many studies to identify or evaluate apps to support medical student learning or any systematic app store searches to identify and evaluate the potential quality of apps aimed at medical students. Such studies would be useful for medical teachers in their role as resource curators [22] so that they can easily compare, identify, and direct students to content-relevant, high-quality apps to support learning. Medical students would also be consumers of such research to find apps that may support self-directed learning. By considering aspects of an app, such as the usefulness of the content being presented, the interactivity of the app in its presentation of content, and its use of methods of learning that increase student satisfaction and interest, such as case-based learning [23], and combining user-centered qualities with technology-centered qualities such as functionality, stability, esthetic appeal, and ease of use [24], we can identify apps that are likely to be effective aids for learning.

We have previously worked with medical students to develop a rubric to evaluate the value of mobile apps to support medical student just-in-time learning [25]. This instrument, the Mobile App Rubric for Learning (MARuL), can be rapidly and easily used by teachers or students to rate the quality of an app and its potential to be useful for learning. MARuL contains 4 categories: teaching and learning measures (n=9), user-centered measures (n=7), professional measures (n=3), and usability measures (n=7). As mobile apps do not yet seem to be widely endorsed or promoted by medical schools to support learning [15,26], MARuL may offer a tool for the faculty to confidently evaluate the quality of apps to support learning [27].

Although the general quality of any health app can be evaluated with the well-established MARS instrument, apps for medical student learning are a subset of health apps that have a specific purpose requiring additional aspects for evaluation. MARuL, though adapting 9 items from MARS, was designed specifically to measure aspects of an app related to its value for medical student learning [25].

### Objectives

This study reports on the use of MARS and MARuL to evaluate apps designed to help medical students develop clinical skills. Clinical skills are a competency that all medical students need to acquire, requiring complex knowledge, psychomotor skills, and integration skills. Good-quality apps could be a useful learning tool for students to acquire these skills. We define clinical skills as any discrete and observable act within the overall process of patient care [27], and for the purposes of this study, we focus on clinical skills required during a traditional doctor-patient interaction. The apps of interest might support the development of history taking, physical examination skills, and patient explanation, which are often assessed in objective structured clinical examinations (OSCEs).

To extend previous work in developing methods of systematic app store search and app evaluation [28] specifically for apps for medical student learning, we aim to do the following:

- Undertake a systematic search of app stores to identify apps available to support clinical skills development by medical students.
- Evaluate the perceived quality of those apps using MARS and the potential value of those apps for student just-in-time learning of clinical skills using MARuL.
- Compare MARS and MARuL as methods for evaluating perceived quality and value of apps for learning.

## Methods

### App Identification

We performed a systematic app search in the New Zealand Apple iOS App Store and Google Play Store between January 15 and February 1, 2019. Search terms were chosen to focus on apps for teaching and learning in health. Three of the authors (TG, GT, and RG) developed the search terms and inclusion and exclusion criteria through preliminary searches and discussion. The final set contained 14 search terms that were searched one at a time (Textbox 1).

**Search terms used in app stores grouped by focus of search term.**

General:

- Clinical skills

Objective Structured Clinical Examination:

- OSCE
- Objective Structured Clinical Examination

History taking:

- Medical history taking
- Clinical history taking
- Patient history

Examinations:

- Medical examination
- Medical exam
- Physical examination
- Physical exam
- Clinical examination
- Clinical exam

Explanation:

- Planning and explaining
- Patient education

### Eligibility Criteria

Apps were initially screened during the search by reading the title and description of the app in the app store. Apps were eligible for inclusion in the review if, in the initial screening, they fulfilled the following 6 inclusion criteria: (1) were available in English; (2) included at least one of the keywords (Textbox 1) in the title or description; (3) included an interactive element requiring some form of input (as deliberate practice with active learning is more effective [14]—to be interactive, an app must require students to perform in some way, eg, by filling in a form, answering questions, or interacting with an image by rotation or other means); (4) their target audience included medical students based on a statement in the app description; (5) supported iOS 8 or later and Android version 5 or later (to include devices in the last 5 years that used these systems); (6) were available for both Android and iOS to ensure student accessibility.

Apps were excluded if they failed to meet the inclusion criteria or if they met any of the following exclusion criteria: (1) priced more than NZ $10 (US $7) for a monthly subscription or as a one-off price; based on a discussion with GT, student research collaborator, and RG, a local leader in medical education and experienced clinician; this was thought to be a reasonable maximum cost that either a student would spend on themselves or an institution would be willing to spend per user; (2) were reference-only apps (passive with no student input, ie, do not require students to interact beyond basic touchscreen requirements such as page turning or pressing play on a video, eg, textbook apps or apps that contain videos of clinical skills being performed that students watch but do not interact with); (3) designed for staff-only use in formative or summative assessment contexts; (4) complemented other software (not stand alone); and (5) required a log in or sign up to be used [29,30] based on a discussion with GT who noted that requiring an initial signup or registration was a barrier to use for most of her student colleagues. These exclusion criteria were based on potential barriers to students’ use or reduced quality of learning for students.

### Data Extraction

A data screening and extraction spreadsheet was developed and refined by 2 researchers (GT and TG) using Airtable [31] before the search. The app name, developer, operating system, reviewer, and whether the app was included or excluded were recorded in the spreadsheet during the initial search and screening of the app store search. Apps were excluded if one of the exclusion criteria was met, and the reason for exclusion was recorded. The iOS store was searched using an iPhone 7 (Apple Inc) and an iPhone 8 using iOS version 12.1.3, and the Google Play Store was searched using 2 Samsung Galaxy J1 Ace phones using Android version 5.1.1.

The app search for the iOS App Store and Google Play Store was completed in parallel but independently by 2 authors (GT and TG). GT and TG then jointly reviewed the apps where there was a lack of agreement. Each discrepancy was discussed, and a final decision was made regarding inclusion or exclusion and the grounds for exclusion. The search and screening are reported based on the PRISMA (Preferred Reporting Items for Systematic Reviews and Meta-Analyses) phase 1 guidelines, modified for app stores and app metadata [32].

### App Rating

All included apps were independently rated by 2 reviewers (JM and SG) with MARS [11] and MARuL [25]. The 2 reviewers were chosen because of their relationship to the student experience. One reviewer is a near peer of medical students, and the other reviewer works extensively with web-based learning for medical education to support student learning. Both MARS and MARuL versions include instructions to consider the target audience for the app, and the individual items of each measure use language keeping the target audience in mind.

First, the 2 reviewers met on videoconference to confirm their understanding of each rubric and its submeasures. They then completed a pilot rating on one excluded app and met on videoconference to discuss their scoring on the items and come to an agreement on how to interpret items that they differed on. The reviewers then independently downloaded and reviewed the included apps in iOS (iPhone 6 Plus and iPhone 6s) and Android (Samsung Galaxy J1 Ace) between November 10 and December 9, 2019. App reviews were completed using the MARS and MARuL in a web-based form (Qualtrics), with data exported to an Excel spreadsheet (Microsoft Office, version 16.40,20081000). The reviewers interacted with each app to fully explore its features before completing the MARS and MARuL. Both category and overall scores on MARS and MARuL for each app were calculated for each reviewer. To measure interrater reliability for MARS and MARuL, intraclass correlation coefficient (ICC) estimates were calculated with their 95% CIs in RStudio [33] based on a single-rating, consistency, two-way mixed effects model [34].

MARS comprises four categories of perceived app quality—engagement, functionality, esthetics, and information—and 1 category of subjective quality. Each category score is the mean of the items, rated on a 5-point Likert-type scale (from 1=inadequate to 5=excellent) within its category. The overall quality score was calculated by taking the mean of the 4 app quality category scores, with a final score ranging from 0 to 5 [11].

MARuL is composed of four categories, each of which receives a score. The category scores are summed together to reach an overall value for the learning score. The MARuL category score is calculated by adding the rating for each item on a 5-point Likert-type scale (0=does not fulfill the item requirements, 1=poorly fulfills the requirements, 2=somewhat fulfills the requirements, 3=mostly fulfills the requirements, and 4=fully meets the requirements) within each category to reach a total score for that category (teaching and learning=36, user-centered=28, professional measures=12, and usability=28). Summing the categories gives the user an overall score of 104. Apps are then categorized by their scoring range (<50=not at all valuable, 51-69=potentially valuable, and >69=probably valuable) [25].

## Results

### App Store Search

**Figure 1:**
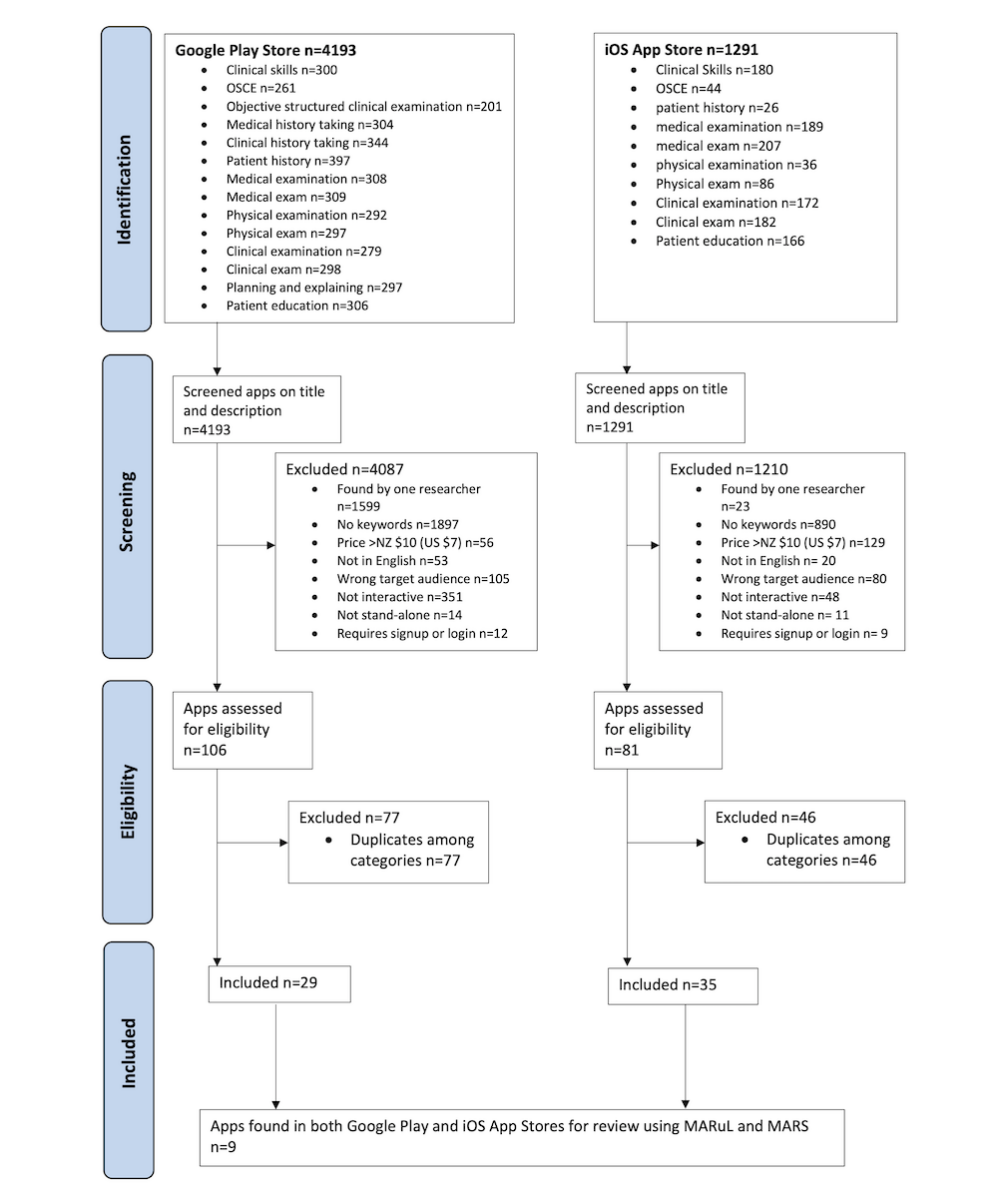
Flowchart for the identification of the Google Play Store and iOS App Store clinical skills apps. MARS: Mobile App Rating Scale; MARuL: Mobile App Rubric for Learning. OSCE: objective structured clinical examinations.

A total of 1291 iOS apps and 4193 Android apps were screened in the iOS App Store and Google Play Store, respectively. Following the app title and description screening, 1210 iOS apps and 4087 Android apps were excluded. Despite using the same search terms, sometimes discrepancies in the results from the search carried out by the 2 reviewers were seen, such as some apps only being found by one reviewer. Only the apps that were found by both reviewers were included in the final sample. We made this decision as our goal for this study was to rate commonly found apps available in both iOS and Android app stores. If the same apps appeared in a search by both a student and staff member, it was felt that they would be commonly located despite any search optimization in use. The two main reasons for exclusion of Android apps were that no keywords were found within the title or description (1897/4087, 46.4%) or that they were only found by one of the 2 researchers (1599/4087, 39.1%). The two main reasons for exclusion of iOS apps were no keywords in the title or description (890/1210, 73.6%), followed by a price greater than NZD $10 (US $7) as a one-off or recurring cost (129/1210, 10.7%). For iOS, 81 apps from the 14 search terms were identified, 35 of which were unique apps. For Android apps, a total of 106 apps were identified, of which 29 were unique. Of the 35 unique iOS apps and 29 unique Android apps, 9 apps were found on both iOS App Store and Google Play Store for inclusion. A search of the Apple Store in the United States using the website fnd.io [35] did not find any further apps that were also available in the international Google Play Store. Figure 1 shows the search and screening process.

### App Characteristics

The characteristics of the apps are summarized in Table 1. The apps ranged in size from 2.7 to 229.2 MB. All apps were free to download except OSCE PASS: Medical Revision, which cost NZD $10 (US $7). Three of the apps, Geeky Medics-OSCE revision; InSimu: The Patient Simulator; and Resuscitation!, had in-app purchases available for additional content. Of the 9 apps, 7 apps were stated to be for medical students and 5 apps specifically focused on clinical skills for OSCE practice.

**Table 1:** Characteristics of the 9 included apps.

App name	Developer	App version	Cost	App size (MB)	Description
CardioSmart; Heart Explorer	American College of Cardiology	3.0 (iOS); 2.3 (Android)	Free	229.2	Interactive 3D heart anatomy and educational pathology videos
Geeky Medics-OSCE^a^ revision	Geeky Medics LTD	2.81 (iOS); 2.46 (Android)	Free; in-app purchases up to NZ $18.99 (US $13.41) on iOS and NZ $29.99 (US $21.18) on Android	76.6	OSCE guides for medical students
InSimu—The Patient Simulator (iOS); InSimu—Diagnose Virtual Clinical Cases (Android)	InSimu	1.7.7 (iOS); 1.8.7 (Android)	Free; in-app purchases up to NZ $499.99 (US $353.09) for lifetime access	51.8	Virtual clinic environment or simulation, work-through diagnosis
OSCE PASS: Medical Revision	Entremed Ltd	1.1 (iOS); 1.0 (Android)	NZ $10 (US $7)	8.3 (iOS); 3.79 (Android)	Written guides and video demonstrations for clinical skills
OSCE Revision for Medical Students (iOS); OSCE Revision (Android)	Matthew Roche	1.2.1 (iOS); 1.1.5 (Android)	Free	10.2	OSCE revision guides with test function
OSCEr	Ahmad Alhashemi (iOS); Essentials of clinical examination (Android)	1.0	Free	22.2	Guides for clinical skills with test and practice options
Pocket PEx: Physical Exam Aid (iOS); Pocket PEx (Android)	Charles Goldberg (iOS); MedEd Apps (Android)	3.1	Free	2.9	Interactive checklists for physical examination
Resuscitation!	EM Gladiators LLC	2.8 (iOS); 2.0 (Android)	Free; in-app purchases up to NZ $16.99 (US $11.99)	82.7	Virtual patient simulator, work-through diagnosis
Patient Education Institute	Olaf Breukhoven (iOS); The Patient Education Institute (Android)	1.2.3 (iOS); 1.2.2 (Android)	Free	2.7	Medical illustrations

^a^OSCE: objective structured clinical examination.

### App Rating

The 9 apps reviewed by the 2 researchers were CardioSmart Heart Explorer; Geeky Medics-OSCE revision; InSimu—The Patient Simulator; OSCE Revision; OSCEr; Pocket PEx: Physical Exam Aid; OSCE PASS: Medical Revision; Patient Education Institute; and Resuscitation! ICC scores for MARuL was ICC (two-way)=0.68 (*P*<.001) and for MARS was ICC (two-way)=0.68 (*P*<.001), indicating moderate reliability (Table 2) [34].

**Table 2:** Interrater reliability scores for the Mobile App Rubric for Learning and Mobile App Rating Scale.

Rating measures	Intraclass correlation	95% CI	*F* test with true value 0	
			*F* test (*df*)	*P* value
MARS^a^	0.677	0.618-0.729	5.2 (367)	<.001
MARuL^b^	0.676	0.621-0.725	5.18 (415)	<.001

^a^MARS: Mobile App Rating Scale.

^b^MARuL: Mobile App Rubric for Learning.

The total app quality mean scores from the MARS evaluation ranged from 2.11 to 3.71 on the 9 iOS apps and 2.21 to 3.79 on the 9 Android apps (Table 3), with lowest scores generally occurring in the engagement and information categories. OSCE PASS: Medical Revision (iOS=3.71; Android=3.79), Geeky Medics-OSCE revision (iOS=3.68; Android=3.74), and CardioSmart Heart Explorer (iOS=3.53; Android=3.53) were the top-scoring apps on iOS and Android.

**Table 3:** Average Mobile App Rating Scale scores from the 2 raters for the 9 apps tested.

Operating system and app name		Total	Engagement	Functionality	Aesthetics	Information	Subjective quality
**Android**							
	OSCE^a^ PASS: Medical Revision	3.79	3.60	4.63	3.83	3.43	3.88
	Geeky Medics-OSCE revision	3.74	3.70	4.38	4.00	3.29	3.88
	CardioSmart Heart Explorer	3.53	3.10	4.38	4.33	3.00	2.00
	Resuscitation!	3.45	3.60	4.38	3.50	2.79	3.63
	OSCEr	3.24	2.90	4.25	3.67	2.71	2.00
	InSimu—The Patient Simulator	3.03	3.20	3.63	3.17	2.50	1.88
	Pocket PEx: Physical Exam Aid	2.76	2.10	4.00	2.67	2.57	1.88
	OSCE Revision for Medical Students	2.39	2.00	2.75	3.00	2.21	1.63
	Patient Education Institute	2.21	1.60	2.75	2.17	2.36	1.00
**iOS**							
	OSCE PASS: Medical Revision	3.71	3.40	4.50	4.00	3.36	3.88
	Geeky Medics-OSCE revision	3.68	3.10	4.75	4.17	3.29	3.75
	CardioSmart Heart Explorer	3.53	3.00	4.25	4.33	3.14	2.38
	Resuscitation!	3.50	3.60	4.50	3.67	2.79	3.63
	OSCEr	3.16	2.90	4.25	3.50	2.57	2.13
	Pocket PEx: Physical Exam Aid	2.84	2.10	4.13	2.67	2.71	1.75
	InSimu—The Patient Simulator	2.76	2.80	3.38	3.33	2.14	1.75
	OSCE Revision for Medical Students	2.66	2.70	3.13	3.00	2.21	1.88
	Patient Education Institute	2.11	1.50	2.63	2.00	2.29	1.00

^a^OSCE: objective structured clinical examination.

The MARuL overall app scores ranged from 21.5 to 73.0 for the 9 iOS apps and 18.0 to 75.0 for the 9 Android apps. Two apps, Geeky Medics-OSCE revision and OSCE PASS: Medical Revision, scored as *probably valuable* in both iOS and Android, and 1 app—Resuscitation!—as *potentially valuable* in both iOS and Android (Table 4). CardioSmart Heart Explorer scored at the low end of the range for *potentially valuable* in Android only. The remaining apps had a MARuL score of less than 50 or *not at all valuable*.

**Table 4:** Average Mobile App Rubric for Learning scores from the 2 raters for the 9 apps tested.

Operating system and app name			Total score out of 104		User-centered score out of 28		Teaching and learning score out of 36		Professional score out of 12		Usability score out of 28
**Android**											
	Geeky Medics-OSCE^a^ revision	75		20		24		8.5		22.5	
	OSCE PASS: Medical Revision	69		20		24		5.5		19.5	
	Resuscitation!	65		19		21		5		20	
	CardioSmart Heart Explorer	54.5		11		12.5		9.5		21.5	
	OSCE Revision for Medical Students	50		8.5		19.5		6.5		15.5	
	OSCEr	47.5		9		15		6		17.5	
	Pocket PEx: Physical Exam Aid	46		7.5		14.5		8.5		15.5	
	InSimu—The Patient Simulator	40.5		8.5		8.5		5.5		18	
	Patient Education Institute	18		1		2		5		10	
**iOS**											
	Geeky Medics-OSCE revision	73		19.5		24		8.5		21	
	OSCE PASS: Medical Revision	73		19.5		25.5		8		20	
	Resuscitation!	66		18.5		22		5.5		20	
	CardioSmart Heart Explorer	49.5		10.5		10.5		10		18.5	
	Pocket PEx: Physical Exam Aid	49		8.5		16		8		16.5	
	OSCEr	48		9		16		6		17	
	InSimu—The Patient Simulator	45		9		8.5		7.5		20	
	OSCE Revision for Medical Students	39.5		5.5		14		6.5		13.5	
	Patient Education Institute	21.5		1.5		2		6		12	

^a^OSCE: objective structured clinical examination.

## Discussion

### Principal Findings

This systematic app store search of the iOS App Store and Google Play Store for apps supporting the development of clinical skills required in the doctor-patient consultation in medical students resulted in the inclusion of 9 relevant apps. The evaluation of the 9 apps—using MARS [11] and MARuL [25]—found only 2 apps that scored highly in fulfilling the quality criteria across both measures of perceived quality for both mobile operating systems, Geeky Medics-OSCE revision and OSCE PASS: Medical Revision. However, each operating system and quality measure identified 3 apps that scored highly in fulfilling the criteria. The top 3 apps as rated by MARS were OSCE PASS: Medical Revision, Geeky Medics-OSCE revision, and CardioSmart Heart Explorer. For MARuL, Geeky Medics-OSCE revision, OSCE PASS: Medical Revision, and Resuscitation! were the top-scoring apps.

MARS and MARuL were designed to measure the perceived quality of apps for different purposes. Although MARS was developed as a method for measuring the perceived quality of a health mobile app for general use purposes [11], MARuL was specifically developed as a measure of the perceived value of a health education app to support student learning [25]. Both measures differentiated between apps of varying quality, as shown by the similarity of their top-ranked apps and the consistency with which they categorized the lowest ranked app, Patient Education Institute, across most of their categories. The similar results for the ranking of apps across the 2 measures indicate that both measures are helpful in characterizing the perceived value or quality of mobile health apps. However, having a category specifically designed to measure teaching and learning allows teachers to use MARuL to measure perceived value for student learning. For example, although CardioSmart Heart Explorer was the third highest rated app using MARS, MARuL rated it at the low end of the category *potentially valuable* on Android devices (54.5), and the iOS version had an overall score of <50 (49.5). Scrutiny of the individual categories of MARuL reveals that CardioSmart Heart Explorer had the third lowest score for teaching and learning in both iOS (10.5) and Android (12.5).

After review using both MARS and MARuL, it was found that the quality of the 9 apps was highly variable. For example, in the MARS evaluations, apps tended to score the highest in the functionality category, followed by esthetics. The scores for engagement and information were the lowest. The engagement category of MARS considers whether the app is fun, interesting, customizable, interactive, and well-targeted to the audience. Similarly, the user-centered category of MARuL considers aspects of the app, such as satisfaction, user experience, and engagement. Of the 9 apps, 6 scored less than half of the possible points in this category. It is concerning that apps consistently scored low in this category, as interest and enjoyment have been found to be strong influencers on students’ persistence of learning [36-38].

One of the criteria for inclusion of apps in our review was the presence of interactivity within the app. Interactivity was evaluated in both MARS and MARuL. Interactivity increases engagement and may stimulate better learning of a topic [14]. Although all the apps reviewed were interactive, the degree of interactivity varied among apps. For example, the app Pocket PEx: Physical Exam Aid had minimal interactivity with checkboxes for each component of a physical exam, whereas the app InSimu had a comprehensive diagnostic scenario with interactivity for each step of the diagnostic process.

Both MARS and MARuL contain a category that includes items on information quality and credibility. Apps performed poorly in this category with only one app, Pocket PEx: Physical Exam Aid, containing easy-to-find references for information. No references were provided for the other apps. This poses a challenge for all types of health apps because of the importance of accurate and evidence-based information [39-41].

As noted earlier, MARuL has a category for the teaching and learning aspects of an app. It includes items on app purpose, pedagogy, capacity to generate learning, quantity of information, relevance to study or course, instructional features, user interactivity, feedback, and efficiency. The highest scoring apps in the teaching and learning category were Geeky Medics-OSCE revision, OSCE PASS, and Resuscitation!, which were also the top-scoring apps overall. Although the teaching and learning category has the highest weighting in the MARuL overall score, and the scoring trend for most apps across the other categories was similar to the teaching and learning category, taking a multidimensional approach to evaluation is important because of the interdependence of the dimensions in measuring value [24]. These findings of variable quality of clinical skills learning apps are consistent with findings from app reviews for patient-centered health-related apps [10,42] and are likely because of the poorly regulated market for mobile health apps.

### Limitations

The app store search was conducted in the New Zealand iOS App Store and Google Play Store. Although this could limit the generalizability of our findings to other countries with different app stores, it should be noted that the Google Play Store is international and a search of the iOS store in the United States using fnd.io [35] did not find any new apps that were included in the Google Play Store. This limitation has been discussed in other app reviews. However, this study specifically focuses on New Zealand medical students; therefore, generalizability is not an immediate concern [43]. The app stores were searched in early 2019. As the rate of change in the app stores is high, it is possible that the apps we originally excluded have now changed enough to be included and other apps may have been removed since the search and review were conducted. The constantly changing nature of apps and their availability in app stores have also been discussed in previous reviews [44,45]. As such, it may prove challenging to keep an up-to-date list of good-quality apps for students to use. The interrater reliability of our MARS and MARuL scores was moderate, which was slightly lower than that described in the MARS and similar to the MARuL development. Although higher reliability might change scoring somewhat, it is unlikely to change our findings, as each individual reviewer identified the same top 3 apps, albeit in a different order, for both MARS and MARuL.

### Next Steps

The results across the 2 measures of app quality indicate the potential convergent and discriminant validity of raters’ perceptions across MARS and MARuL. Further research to develop the construct validity of these 2 measures by using student outcome data with regard to highly rated apps will help to confirm their usefulness in their respective areas of focus.

### Conclusions

This systematic search for and evaluation of clinical skills mobile apps for perceived general quality and value for learning has highlighted the importance of using a fit-for-purpose measure of quality or value of mobile apps. The findings suggest that both MARS and MARuL instruments are useful and somewhat complementary. This study also highlights the variable quality of health-related education apps, likely because of the lack of regulation of health apps, in the iOS App Store and Google Play Store. However, Geeky Medics-OSCE revision and OSCE PASS are examples of how good practice in the development of apps can lead to quality apps for learning.

## Abbreviations

ICC: intraclass correlation coefficient
MARS: Mobile App Rating Scale
MARuL: Mobile App Rubric for Learning
OSCE: objective structured clinical examination
PRISMA: Preferred Reporting Items for Systematic Reviews and Meta-Analyses
